# The Spectrum of Antimicrobial Activity of Cyadox against Pathogens Collected from Pigs, Chicken, and Fish in China

**DOI:** 10.3390/antibiotics10020153

**Published:** 2021-02-03

**Authors:** Muhammad Kashif Maan, Zhifei Weng, Menghong Dai, Zhenli Liu, Haihong Hao, Guyue Cheng, Yulian Wang, Xu Wang, Lingli Huang

**Affiliations:** National Reference Laboratory of Veterinary Drµg Residues/MOA Key Laboratory of Food Safety Evaluation, Huazhong Agricultural University, Wuhan 430070, China; kashif.maan@uvas.edu.pk (M.K.M.); hzauwengzhifei@163.com (Z.W.); daimenghong@mail.hzau.edu.cn (M.D.); liuzhli009@mail.hzau.edu.cn (Z.L.); haohaihong@mail.hzau.edu.cn (H.H.); chengguyue@mail.hzau.edu.cn (G.C.); wangyulian@mail.hzau.edu.cn (Y.W.); wangxu@mail.hzau.edu.cn (X.W.)

**Keywords:** cyadox, antimicrobial activity, pathogenic bacteria, clinical breakpoints

## Abstract

Cyadox has potential use as an antimicrobial agent in animals. However, its pharmacodynamic properties have not been systematically studied yet. In this study, the in vitro antibacterial activities of cyadox were assayed, and the antibacterial efficacy of cyadox against facultative anaerobes was also determined under anaerobic conditions. It was shown that *Clostridium perfringens* and *Pasteurella multocida* (MIC = 0.25 and 1 μg/mL) from pigs, *Campylobacter jejuni* and *Pasteurella multocida* from poultry, *E. coli*, *Streptococcus* spp., and *Flavobacterium columnare* from fish were highly susceptible to cyadox (MIC= 1 and 8 μg/mL). However, *F. columnare* has no killing effect for drug tolerance. Under in vitro anaerobic conditions, the antibacterial activity of cyadox against most facultative anaerobes was considerably enhanced Under anaerobic conditions for the facultative anaerobes, susceptible bacteria were *P. multocida*, *Aeromonas spp*. (including *A. hydrophila*, *A. veronii*, *A. jandaei*, *A. caviae*, and *A. sobria*, excluding *A. punctata*), *E. coli*, *Salmonella* spp. (including *S. choleraesui*, *S. typhimurium*, and *S. pullorum*), *Proteus mirabilis*, *Vibrio fluvialis*, *Yersinia ruckeri*, *Erysipelothrix*, *Acinetobacter baumannii*, and *Streptococcus agalactiae* (MICs were 0.25~8 μg/mL, MBCs were 1–64 μg/mL). Intermediate bacteria were *Enterococcus* spp. (including *E. faecalis* and *E. faecium*), *Yersinia enterocolitica*, and *Streptococcus* spp. (MICs mainly were 8~32 μg/mL, MBCs were 16~128 μg/mL). This study firstly showed that cyadox had strong antibacterial activity and had the potential to be used as a single drug in the treatment of bacterial infectious diseases.

## 1. Introduction

Cyadox is a synthetic compound belonging to quinoxaline-1,4-dioxides, which are widely used as an antibacterial agent with a broad spectrum of antimicrobial activity and growth promoters in veterinary medicine [1]. Compared with the other members of quinoxalines such as carbadox and olaquindox, the cyadox is safer [2,3,4,5] according to the long term toxicity test, a subchronic oral toxicity test, and a phototoxicity test of cyadox in previous studies [6] and can promote the growth of different animals with more obvious effects such as better growth-enhancing functions in food-producing animals including fish, goats, pigs and poultry with less toxic effects than olaquindox, when used as feed additive [7] in animal feed. Moreover, further studies have demonstrated that cyadox was better as a growth promotor if compared with carbadox and olaquindox [8]. Since carbadox and olaquindox have been banned or limited to be used in food animals due to their toxicities, making cyadox as a substitution product having a capacious prospect in animal husbandry and aquaculture. Cyadox has excellent pharmacokinetic characteristics. Previous studies have shown the distribution and metabolism of Cyadox in swine, and six major metabolites were identified as follows: Disdesoxy- Cyadox (Cy1), Cyadox 4-monoxide (Cy2), N-decyanoacetyl Cyadox (Cy4), Quinocaline-2-carboxylic acid (Cy6), 11, 12-dihydro-bisdesoxycaydox (Cy9), 2-hydromethyl-quinoxaline (Cy12). To fully reflect the pharmacodynamic of cyadox, it is necessary to detect the antibacterial activity of cyadox and its metabolites.

However, there are few studies on the pharmacodynamics of cyadox at present. As a potent antimicrobial agent, Cyadox had been proved to have a wide spectrum of activity against many pathogenic bacteria of pigs, poultry, and fish [9]. In vivo, cyadox reduces diarrhea frequencies of different animals and prevents *E. coli* infection in piglets and broilers [10]. It has high antimicrobial activity in vitro against *E. coli* under anaerobic conditions. MIC values for cyadox in MHB (Mueller–Hinton broth) against *E. coli* were 1 to 4 μg/mL [11]. Some studies showed that cyadox could promote the growth of swine, chicken, and fish [3,12]. However, there are only limited data on the prophylactic schedule in piglets. At present, there is not a scientifically validated dosage for treating *E. coli* diarrhea. 

However, the results of previous studies were not sufficient to explain the antimicrobial characteristics of cyadox. Hence, there is a need for a further and complete study to build up the antimicrobial spectrum using the standard method of Clinical and Laboratory Standards Institute (CLSI) approved by the FDA.

The purpose of this study was to evaluate the minimum inhibitory concentration (MIC) and minimal bactericidal concentration (MBC) of cyadox in vitro against different species of bacteria from pigs, poultry, and fishes, most of which were enteric pathogens, and compare the antimicrobial spectrum of cyadox with other commonly used antimicrobial agents. Under anaerobic conditions, the antimicrobial activity of some quinoxalines were different as compared to cyadox because cyadox exhibits much stronger activity in the absence of oxygen [10]. therefore, cyadox may be active against facultative anaerobes under anaerobic conditions. Based upon systematic toxicological and microbiological safety evaluations, cyadox shows much lower toxicity and higher safety than other well-known quinoxalines such as olaquindox and carbadox, which have been banned or strictly limited in their use in food-producing animals because of their potential toxicities [13]. However, it is hopeful that cyadox would be developed as a replacer of olaquindox and carbadox with greater safety and excellent antimicrobial activity. Based on the related guidelines and standards of the Clinical and Laboratory Standards Institute (CLSI), we determined the in vitro antibacterial activities of cyadox and established the antimicrobial spectrum of cyadox comprehensively and systematically in pathogenic bacteria from swine, chicken, and fish in the present study. The deep knowledge about the pharmacodynamics of cyadox will lay a solid foundation for the application of cyadox as a new veterinary drug.

## 2. Materials and Methods 

### 2.1. Bacteria

Standard strains of *E. Coli, Pasteurella multocida*, *Salmonella, Erysipelothrix*, *Streptococcus*, *Enterococcus* spp., and *Clostridium perfringen* were obtained from China Veterinary Culture Collection Center (CVCC) and American Type Cell Culture (ATCC). Pathogenic bacteria (including 7 quality control strains and 4 testing strains *Aeromonas veronii*, *Pseudomonas pyocyanea*, *Salmonella typhimurium*, and *Proteus mirabilis*) were obtained directly from the ATCC and MicroBiologics (St Cloud, MN, USA). Other clinical isolates of pigs and chickens (*Escherichia coli* 9 strains, *Pasteurella multocida* 1 strain, *Salmonella pullorum* 8 strains, *Staphylococcus aureus* 3 strains, *Streptococcus* spp. 2 strains) were obtained from State Key Laboratory of Agricultural Microbiology, Huazhong Agricultural University, Wuhan, China. Fish pathogenic bacteria (*Yersinia ruckeri* SC90-2-4, *Aeromonas hydrophila* XS91-4-1, *Aeromonas jandaei* F30-3, *Aeromonas caviae* DMA1-A, *Aeromonas sobria* CR79-1-1, *Aeromonas punctata* 58-20-9, *Edwardsiella ictaluri* HSN-1, *Vibrio fluvialis* WY91-24-3, *Flavobacterim columnare* G4, *Pseudomonas fluorescent* W81-11 and 56-12-10, *Streptococcus agalactiae* XQ-1, and the 4 strains of *Mycobacterium tuberculosis*) were derived from numerous laboratories of State Key Laboratory of Freshwater Ecology and Biotechnology, Institute of Hydrobiology, Chinese Academy of Sciences. Other fish pathogens (*Escherichia coli* 1 strains, *Aeromonas hydrophila* 4 strains, *Aeromonas sobria* 3 strains, *Acinetobacter baumannii* 1 strains, *P. fluorescent* 8 strains, *Staphylococcus aureus* 2 strains) were obtained from the College of Fisheries, Huazhong Agricultural University, Wuhan, China. All the strains were stored at −70 ℃ in 20% skimmed milk. All the bacteria were inoculated at least twice on MH (Mueller Hinton) agar growth media prior to testing.

### 2.2. Study Drug and Susceptibility Testing

Cyadox powder (purity percent is ≥98%) was synthesized by the Institute of Veterinary Pharmaceutics (Huazhong Agricultural University, Wuhan, China). For the preparation of the working solution for MIC determination desired amount of cyadox was dissolved in dimethyl sulfoxide (DMSO) at the concentration of 1280 μg/mL as a stock solution. For MIC (Minimum inhibitory Concentration) determination, each bacterial strain was cultured to a logarithmic phase to obtain the turbidity of the 0.5 McFarland standard and then was diluted 100 times with MH broth to obtain a density of 1 × 10^6^ CFU/mL which was used as the inoculum suspension. MIC was defined as the minimum concentration of compound that resulted in no visible growth. MIC determination was performed by the microbroth dilution method according to the CLSI (Clinical and Laboratory Standards Institute, formerly NCCLS) guidelines. The test was performed in a 96-well microtiter plate in a final volume of 100 μL. Each well was inoculated with serially diluted antimicrobial agents and the inoculum suspension (1:1 *v/v*). Different inoculation conditions for different bacteria isolated from livestock and poultry were used for MIC determination. Nonfastidious bacteria (*Escherichia coli*, *Salmonella* spp., *Yersinia* spp. *Proteus mirabilis*, *Pseudomonas* spp., *Staphylococcus aureus, Enterococcus* spp.) were cultured in CAMHB (cation-adjusted Mueller-Hinton broth) at 37 ℃ for 16–20 h according to the CLSI guidelines. Fastidious organisms (*Pasteurella* spp., *Streptococcus* spp., and *Erysipelothrix* spp.) were cultured in the media of CAMHB+LHB (cation-adjusted Mueller–Hinton broth supplemented with 2.5% lysed horse blood) for 18–20 h at 37 °C. Microaerophilic bacteria *Campylobacter jejuni* were cultured in CAMHB+LHB at 42 °C for 24 h under 10% CO_2_. Anaerobic bacteria, such as *Clostridium perfringens* were cultured in Brucella broth under 80% N_2_-10% CO_2_-10% H_2_ at 37 °C for 24 h. The inoculation conditions for the bacteria isolated from fish were set according to the CLSI guidelines at a temperature of 28 °C. *Vibrio fluvialis* was cultured in CAMHB with 1% NaCl for 24 h; *E. ictaluri* was cultured in CAMHB for 48 h; *Flavobacterim columnare* was cultured in CAMHB diluted for 24 h; *Streptococcus agalactiae* was cultured in CAMHB supplemented with 2.5% lysed horse blood for 24 h. *E. coli*, *F. columnare*, *Aeromonas* spp. (including *A. hydrophila*, *A. veronii*, *A. jandaei*, *A. caviae*, *A. sobria*, and *A. punctata*), *V. fluvialis*, *A. baumannii*, and *Y. ruckeri* isolated from fish were cultured in CAMHB for 24 h. *Mycobacterium tuberculosis* was cultured on Lowenstein–Jensen medium (LJ) solidified by coagulation at 83 °C for 40 min and incubated at 37 °C [14]. Quality control was monitored using *Escherichia coli* ATCC 25922, *Enterococcus faecalis* ATCC 29212, *Staphylococcus aureus* ATCC 29213, *Streptococcus pneumoniae* ATCC 49619, *Campylobacter jejuni* ATCC 33560, *Bacteroides fragilis* ATCC 25285, and *Bacteroides thetaiotaomicron* ATCC 29741. 

MBC (Minimal Bactericidal Concentration) was determined according to the document M26-AE of CLSI. The lowest concentration of antimicrobial agent that killed ≥99.9% of the starting inoculum was defined as the MBC endpoint. The double diluted inoculum suspension and 10 μL broth from 96-well with no visible growth above the MIC after 24 h incubation on MH agar, incubated for one or two nights and counted for colony, respectively, and calculated for the MBC further.

MICs of the facultative anaerobes tested under anaerobic conditions were determined according to the defined methodology of CLSI with little change in the anaerobic environement (80% N_2_—10% CO_2_—10% H_2_). Bacteria for colony counting and MBC testing were cultured under aerobic condition.

All the experiments were performed in 3 replicates along with the quality control strains to ensure the accuracy of results. 

### 2.3. Data Processing 

For analytical purposes, the bacteria were grouped into species or genus groups. The calculation included in MIC_50_ (MBC at which 50% of the strains are inhibited), MBC_90_ (MBC at which 90% of the strains are inhibited), MBC_50_ (MBC at which 50% of the strains are killed), MBC_90_ (MBC at which 90% of the strains are killed), and the MBC/MIC ratios were calculated to determine the presence or absence of tolerance. MIC50, MBC50, MIC90, and MBC90 were calculated by using SPSS software. The breakpoint was set in present study as follow: susceptible, MIC_90_ ≤ 8 μg/mL; intermediate, 16 μg/mL ≤ MIC_90_ ≤ 32 μg/mL; resistant, MIC_90_ ≥ 64 μg/mL. Tolerance was defined as an MBC/MIC ratio of ≥32 or an MBC/MIC ratio of ≥16 when the MBC was greater than or equal to the MIC resistance breakpoint. 

## 3. Results 

### 3.1. Susceptibility of Pig Pathogens to Cyadox

Under CLSI standard conditions, the MIC and MBC of cyadox against *Clostridium perfringen* were 0.5~1 μg/mL, which were more susceptible and stronger than that of other antibacterial agents. The cyadox was much more effective against *Pasteurella multocida*, *Salmonella choleraesui*, *Erysipelothrix*, and *Streptococcus* than olaquindox but weaker than chlortetracycline. *Streptococcus* were found to be resistant to chlortetracycline. Under anaerobic conditions for facultative anaerobes, the antibacterial activity of cyadox was enhanced by 4–6 times in *E. coli*, *Pasteurella multocida*, *Salmonella choleraesuis*, and *Erysipelothrix*. Compared with controls, the antibacterial activity of cyadox was stronger than that of other antibacterials against *Escherichia coli*; the actions of cyadox were stronger than or similar to that of olaquindox and weaker than that of chlortetracycline against other bacteria (Table 1). 

### 3.2. Susceptibility of Poultry Pathogens to Cyadox 

Following CLSI standards conditions, the most susceptible bacteria forcyadox were *C. jejuni* and *C. perfringen* with the MICs and MBCs were 0.25~1 μg/mL and 1 μg/mL, respectively. While *E. faecalis* and *E. faecium* were resistant against cyadox. Under anaerobic conditions for facultative anaerobes, the antibacterial activity of cyadox was enhanced by 4~16 times in *S. pullorum*, *E. coli*, and *Enterococcus* spp., which indicated an inclined effect of cyadox against these bacteria. Compared with controls, under the two incubating conditions, the antibacterial actions of cyadox were stronger than that of other antibacterial agents against *E. coli* and *C. perfringen*, and the action of cyadox was stronger than or similar to that of olaquindox but weaker than that of chlortetracycline against other bacteria (Table 2).

### 3.3. Susceptibility of Fish Pathogens to Cyadox 

*E. coli* showed a susceptible effect to cyadox with the MIC and MBC was 1 μg/mL and 16 μg/mL, respectively. For *F. columnare*, cyadox and sulfadimidine showed only an inhibitory effect but not a bactericidal effect. Under anaerobic conditions for facultative anaerobes, the antibacterial activity was enhanced by 8~256 times in *Aeromonas* spp. (included *A. hydrophila*, *A. veronii*, *A. jandaei*, *A. caviae*, and *A. sobria*, excluding *A. punctata*), *V. fluvialis*, *A. baumannii*, and *Y. ruckeri*. MICs and MBCs of *Aeromonas* spp. (excluded *Aeromonas punctata*), *V. fluvialis*, and *Y. ruckeri* were declined to 0.5~2 μg/mL and 1~8 μg/mL. Compared with controls, the antibacterial activity of cyadox were stronger than that of other antibacterial agents against *E. coli*. For *Mycobacterium tuberculosis*, the action of cyadox was stronger or similar to sulfadimidine but weaker than that of chlortetracycline against other bacteria except for *A. baumannii* (Table 3).

### 3.4. Susceptibility of Other Pathogens to Cyadox 

The results of antimicrobial susceptibility of cyadox against pathogenic bacteria isolated from humans and animals were listed in (Table 4). The antibacterial action of cyadox was stronger than that of other antibacterial agents against *S. typhimurium*, *Y. enterocolitica*, and *P. mirabilis* which was stronger than sulfonamide but weaker than chlortetracycline. Under anaerobic conditions, the antibacterial activity was enhanced by 8 times in *Proteus mirabilis*. 

## 4. Discussion

Clinical breakpoints for quinoxalines have not been established by CLSI yet [15]. This study defines the clinical breakpoints for cyadox according to the antibacterial activities of cyadox and the antibiogram of olaquindox. Cyadox has a good effect against *E. coli* in vitro (MIC_90_ under anaerobic condition was 4 μg/mL in this test). The susceptible bacteria of olaquindox including gram-negative bacteria (*P. multocida*, *E. coli*, *S. choleraesui*, *Shigella* spp., and *Proteus* spp.) and gram-positive bacteria (*Staphylococci*), MIC_90_ of these bacteria under anaerobic condition were 8 μg/mL in this study. In addition, isolates were considered to be tolerant to antimicrobial agents that were known to be bactericidal but that do not show a killing effect.

The antimicrobial effect of cyadox against pathogens isolated from pigs and poultry was similar in vitro. Under standard conditions, susceptible bacteria for cyadox were *C. perfringen*, *C. jejuni*, and *P. multocida*. Intermediate bacteria were *Salmonella* spp. (including *S. choleraesui*, *S. typhimurium*, *S. pullorum*), *E. coli*, *Y. enterocolitica*, *P. mirabilis*, *Erysipelothrix*, *S. aureus*, and *Streptococcus* spp. The susceptibility of *C. perfringens* against cyadox was similar to previous studies conducted by [11]. Resistant bacteria were *P. pyocyanea*, *E. faecalis*, and *E. faecium*. Under anaerobic conditions, susceptible bacteria were *P. multocida*, *E. coli*, *Salmonella* spp., *P. mirabilis*, and *Erysipelothrix*, intermediate bacteria were *Y. enterocolitica*, *Streptococcus* spp, *E. faecalis*, and *E. faecium*, while *P. pyocyanea* was resistant bacterium against cyadox. However, cyadox showed growth inhibition with a ≥16-fold against *Salmonella* spp. and *Erysipelothrix* spp. While compared with other drugs, *Salmonella* spp., *Erysipelothrix* spp., and *Streptococcus* spp. have a high tolerance against chlortetracycline, and *C. perfringen* has a high tolerance against olaquindox.

Cyadox showed broad-spectrum activity against pathogens isolated from fish. Under standard conditions, susceptible bacteria for cyadox were *E. coli* and *F. columnare*, but for the later bacterium cyadox has no killing effect. Intermediate bacteria were *Yersinia ruckeri*, *Staphylococcus aureus*, *S. agalactiae*, and *Mycobacterium tuberculosis*. Nonsusceptible bacteria were *P. fluorescent*, *A. baumannii*, *Aeromonas* spp., *E. ictaluri*, and *V. fluvialis*. Under anaerobic conditions, susceptible bacteria were *Aeromonas* spp. (excluded *Aeromonas punctata)*, *V. fluvialis*, and *Y. ruckeri*, intermediate bacteria were *S. agalactiae* and *A. baumannii*, nonsusceptible bacteria were *P. fluorescent*, *A. punctata*, and *E. ictaluri*.

Compared with the source of pathogenic bacteria, the antimicrobial spectrum of cyadox against pigs and poultry in vitro was similar. The antimicrobial effect of cyadox against different serotypes or serogroup in the same species was almost similar, but the MICs of some *Streptococcus* isolated in recent years increased, which suggests no cross-resistance between quinoxalines except *Streptococcus*. The antimicrobial susceptibility of bacteria isolated from fish was different from that of bacteria from non-fish source, which mainly because the incubation temperature was different. Under aerobic conditions, the MICs and MBCs of cyadox in *E. coli* were the same as or higher than that of olaquindox, which means that the antibacterial effect of cyadox against *E. coli* is not as good as olaquindox in vitro. However, the effect of cyadox on the antibacterial activity in vitro was as good as that of olaquindox against *E. coli* infection demonstrated its activity under aerobic conditions [10]. Maybe it can turn to seek an answer from the bactericidal activity of cyadox and olaquindox under anaerobic conditions. The effect under anaerobic conditions is closer to that of the intestinal tract condition than that under the aerobic condition [16]. 

The antimicrobial activity of cyadox for most facultative anaerobes was significantly better in anaerobic conditions than in aerobic conditions, the sensitivity of control drug chlortetracycline and olaquindox were significantly improved as compared to Bacitracin-zinc and sulfadimidine. In this test, the MICs of *Escherichia coli* and *Salmonella* spp. under CLSI condition and anaerobic condition were in accordance with that reported previously [11]. The difference in antibacterial activity of some quinoxalines under anaerobic and aerobic conditions may be due to some free radicals [17]. The antibacterial mechanism may be similar to quindoxin. There was no evidence has been found for binding of quindoxin to DNA [18]. It suggested that some free radicals responsible for the lethal effect of quindoxin, and the free radicals were generated always accompanied by a reduction of the drug and occurred only under anaerobic conditions [18].

Usually, the treatment effect in vivo can be predicted by the results in vitro [19]. Cyadox exhibited excellent in vitro activity across an extended spectrum of bacteria, encompassing all major pathogens with clinical relevance of intestines infections in pigs, poultry, and fish [20]. Cyadox used as an antimicrobial growth promoter has good potential for disease resistance, which needs further clinical trials to validate especially in fish production. In addition, the better antimicrobial activity under anaerobic conditions provides a new aspect of investigation for further clinical studies. 

In conclusion, this study has determined MICs of cyadox against pathogens from swine, chicken, and fish and established the antibacterial spectrum of activity of cyadox. It is shown that cyadox has a good antibacterial activity which is better than other quinoxaline derivatives. Under in vitro anaerobic conditions, the antibacterial activity of cyadox against most facultative anaerobes is considerably better which demonstrated that cyadox is an active compound in anaerobic conditions, which provides a reasonable theoretical foundation for the clinical application of cyadox. The overall in vitro results provide predictive evidence that cyadox has high antibacterial activity that can be used alone even though we are hunting appropriate medications for drug combinations.

## Figures and Tables

**Table 1:** Antimicrobial susceptibility of cyadox and controls against pathogens isolated from pigs (unit: μg/mL).

Number	Serotype	Cyadox				Chlortetracycline				Olaquindox				Dimethyl Sulfoxide	
		MIC_S_	MBC_S_	MIC_N_	MBC_N_	MIC_S_	MBC_S_	MIC_N_	MBC_N_	MIC_S_	MBC_S_	MIC_N_	MBC_N_	MIC_S_	MIC_N_
**G ^−^**															
***E. coil***															
CVCC196	O8:K87,K88ac	32	64	2 (16)	8 (8)	4	32	0.5 (8)	8 (4)	16	128	4 (4)	8 (16)	128	128
CVCC220	O101:K32	32	128	2 (16)	8 (16)	64	128	16 (4)	32 (4)	16	128	4 (4)	8 (16)	128	128
CVCC216	O8:K87,K88ad	32	64	4 (8)	16 (4)	32	32–64	4 (8)	32 (1–2)	32	128	8 (4)	16 (8)	128	128
CVCC223	O141:K99	32	64	1 (32)	8 (8)	64	64	64 (1)	>64 (1)	16	>128	2 (8)	16 (>8)	>128	128
CVCC224	O149:K91,K88ac	32	64	2 (16)	16 (4)	64	64	64 (1)	>64 (1)	32	128	4 (8)	8 (16)	128	128
CVCC1500	O149:K88ac	32	128	2 (16)	16 (8)	64	128	64 (1)	64 (2)	32	128	8 (4)	16 (8)	128	64
CVCC1502	O9:K88	32	128	4 (8)	16 (8)	64–128	128	32 (2–4)	64 (2)	32	64	4 (8)	16 (4)	64	128
CVCC1513	O101:K99	32	>128	1 (32)	8(>16)	64	128	32 (2)	64 (2)	16	128	2 (8)	8 (16)	128	128
CVCC1519	O139	32	>128	2 (16)	32 (>4)	64	128	32 (2)	64 (2)	32	128	8 (4)	16 (8)	128	128
CVCC1514	O45:K99	32	128	0.5 (64)	16 (8)	64	128	8 (8)	64 (2)	8	16	2 (4)	8 (2)	128	128
		MIC_50_ = 32, MBC_50 _ = 128		MIC_N50 _ = 2, MBC_N50 _ = 16		MIC_50 _ = 64, MBC_50 _ = 128		MIC_50 _ = 32, MBC_50 _ = 64		MIC_50 _ = 16, MBC_50 _ = 128		MIC_50 _ = 4, MBC_50 _ = 8			
		MIC_90_ = 32, MBC_90_ > 128		MIC_90 _ = 4, MBC_90 _ = 16		MIC_90 _ = 64, MBC_90 _ = 128		MIC_90 _ = 64, MBC_90 _ = 64		MIC_90 _ = 32, MBCjhjhh_90 _ = 128		MIC_90 _ = 8, MBC_90 _ = 16			
		MBC_50_/MIC_50 _ = 4		MBC_50_/MIC_50 _ = 8		MBC_50_/MIC_50 _ = 2		MBC_50_/MIC_50 _ = 2		MBC_50_/MIC_50 _ = 8		MBC_50_/MIC_50 _ = 2			
***P. multocida***															
CVCC430	B:2,5	16	16			0.5	2			32	64			128	
CVCC432	A:1	8	16			0.5	2			16	32			128	
CVCC433	D:7	2	4	0.13 (16)	0.5 (8)	0.5	2	0.06 (8)	0.5 (4)	4	8	0.5 (8)	4 (2)	64	128
CVCC435	A:1	16	32	0.25 (64)	1 (32)	0.5	2	0.03 (16)	0.25 (8)	16	64	2 (8)	4 (16)	128	64
CVCC436	A:1	8	64	0.25 (32)	1 (32)	0.5	2	0.03 (16)	0.25 (8)	16	64	1 (16)	4 (16)	128	64
CVCC437	A:6	4	8	1 (4)	2 (4)	0.5	4	0.03 (16)	0.25(16)	8–16	32	1(8–16)	4 (8)	128	128
CVCC438	A:1	4	8	0.25 (16)	1 (8)	1	4	0.03 (32)	0.5 (8)	2	8	1 (2)	4 (2)	128	128
CVCC439	D:3	8	16			0.5	2–4			8–16	32			128	
CVCC440	A:6	8	16	0.5 (16)	2 (8)	0.25–0.5	2	0.06(4–8)	0.25 (8)	8	32	2 (4)	8 (4)	128	128
CVCC441	B:2,5	4	8	0.5 (8)	2 (4)	0.5	4	0.03 (16)	0.25(16)	8–16	16	1(8–16)	4 (4)	128	64
CVCC443	A:1	8	16	0.5 (16)	2 (8)	0.5	2	0.03 (16)	0.25 (8)	8	16	1 (8)	4 (4)	128	64
CVCC444	A:1	8	16			0.5	4			16	32			128	
CVCC446	B:2,5	8	16	0.25 (32)	2 (8)	0.5	2	0.03 (16)	0.25 (8)	16	32	2 (8)	4 (8)	128	64
		MIC_50 _ = 8, MBC_50 _ = 16		MIC_50 _ = 0.25, MBC_50 _ = 2		MIC_50 _ = 0.5, MBC_50 _ = 2		MIC_50 _ = 0.03, MBC_50 _ = 0.25		MIC_50 _ = 16, MBC_50 _ = 32		MIC_50 _ = 1, MBC_50 _ = 4			
		MIC_90 _ = 8, MBC_90 _ = 32		MIC_90 _ = 0.5, MBC_90 _ = 2		MIC_90 _ = 1, MBC_90 _ = 4		MIC_90 _ = 0.06, MBC_90 _ = 0.25		MIC_90 _ = 16, MBC_90 _ = 64		MIC_90 _ = 2, MBC_90 _ = 4			
		MBC_50_/MIC_50 _ = 2		MBC_50_/MIC_50 _ = 8		MBC_50_/MIC_50 _ = 4		MBC_50_/MIC_50 _ = 8		MBC_50_/MIC_50 _ = 2		MBC_50_/MIC_50 _ = 4			
***S. choleraesuis***															
CVCC503	6,7:C:1,5	8	>128	1 (8)	32 (>4)	4	64	1 (4)	64 (1)	32	>128	2 (16)	4 (>32)	>128	128
CVCC504	6,7:C:1,5	32	>128	4 (8)	64 (>2)	8	64	2 (4)	64 (1)	8	>128	2 (4)	8 (>16)	>128	128
		MBC/MIC > 16		MBC/MIC = 16–32		MBC/MIC = 8–16		MBC/MIC = 32–64		MBC/MIC > 16		MBC/MIC = 2–4			
G ^+^															
***Erysipelothrix***															
	1a	32	128	4 (8)	32 (4)	0.5	32	0.06 (8)	2 (16)	32	>128	32 (1)	64 (>2)	>128	128
	8	32	>128	4 (8)	64 (>2)	0.5	32	0.06 (8)	2 (16)	32	128	32 (1)	64 (2)	128	128
CVCC1293	5	32	>128	4 (8)	64 (>2)	0.5	16	0.06 (8)	2 (8)	32	128	32 (1)	64 (2)	128	128
		MIC_50 _ = 32, MBC_50_ > 128		MIC_50 _ = 4, MBC_50 _ = 64		MIC_50 _ = 0.5, MBC_50 _ = 32		MIC_50 _ = 0.06, MBC_50 _ = 2		MIC_50 _ = 32, MBC_50 _ = 128		MIC_50 _ = 32, MBC_50 _ = 64			
		MBC_50_/MIC_50_ > 4		MBC_50_/MIC_50 _ = 16		MBC_50_/MIC_50 _ = 64		MBC_50_/MIC_50 _ = 32		MBC_50_/MIC_50 _ = 4		MBC_50_/MIC_50 _ = 2			
***Streptococcus* spp.**															
CVCC606	Gram-R group	32	64	16 (2)	64 (1)	8	128	0.25 (32)	8 (16)	>128	>128	64 (>2)	128 (>1)	128	128
CVCC607	Gram-R group	32	64	32 (1)	64 (1)	0.25	>128	0.06 (4)	1(>128)	>128	>128	64 (>2)	128 (>1)	>128	128
CVCC608	Gram-S group	32	64	32 (1)	64 (1)	0.25	>128	0.06 (4)	1(>128)	>128	>128	64 (>2)	128 (>1)	>128	128
CVCC609	Gram-S group	16	32	16 (1)	32 (1)	0.13	128	0.06 (2)	1 (128)	>128	>128	64 (>2)	128 (>1)	128	128
sc19	Capsule-Ⅱtype	64	>128	32 (2)	128 (>1)	64	128	16 (4)	16 (8)	>128	>128	64 (>2)	128 (>1)	128	128
sc109	Capsule-Ⅱtype	128	>128	64 (2)	128 (>1)	32	>128	0.25(128)	2 (>64)	>128	>128	64 (>2)	128 (>1)	>128	>128
		MIC_50 _ = 32, MBC_50 _ = 64		MIC_50 _ = 32, MBC_50 _ = 64		MIC_50 _ = 0.25, MBC_50 _ = 128		MIC_50 _ = 0.06, MBC_50 _ = 1		MIC_50_ > 128, MBC_50_ > 128		MIC_50 _ = 64, MBC_50 _ = 128			
		MBC_50_/MIC_50 _ = 2		MBC_50_/MIC_50 _ = 2		MBC_50_/MIC_50 _ = 512		MBC_50_/MIC_50 _ = 4				MBC_50_/MIC_50 _ = 2			
***C. perfringens***															
CVCC1125	A	1	1			0.03	0.06			1	128			128	
CVCC1160	C	0.5–1	1			8	8			1	128			128	
		MBC/MIC = 1–2				MBC/MIC = 1–2				MBC/MIC = 128					

Note: (1) The lower symbol “_S_” denotes the result was under CLSI condition, and “_N_” denotes the result was under anaerobic condition. (2) Values in brackets are the multiple drop of MIC under anaerobic conditions from aerobic conditions. “+” denotes gram-positive and “−” denotes gram-negative.

**Table 2:** Antimicrobial susceptibility of cyadox and controls against pathogens isolated from poultry (unit: μg/mL).

Number	Serotype	Cyadox				Chlortetracycline				Bacitracin Zinc					Dimethyl Sulfoxide	
		MIC_S_	MBC_S_	MIC_N_	MBC_N_	MIC_S_	MBC_S_	MIC_N_	MBC_N_	MIC_S_		MBC_S_	MIC_N_	MBC_N_	MIC_S_	MIC_N_
**G ^−^**																
***E. coil***																
CVCC1496	O139:K^+^	32	128	2 (16)	32 (4)	64	64	16 (4)	64 (1)	>128	>128	>128 (1)		>128 (1)	>128	>128
C84010	O1	16	64	1 (16)	8 (16)	32	64	8 (4)	64 (1)	128	>128	128 (1)		>128 (1)	>128	>128
E-O1	O1	16	64			32	128			>128	>128				>128	
E-O2	O2	32	64			32	128			>128	>128				>128	
E-O24	O24	64	>128			128	>128			>128	>128				>128	
E-O78	O78	32	64			64	64			>128	>128				>128	
W1		64	128			64	128			>128	>128				>128	
W2		32	64			16	32			>128	>128				>128	
W3		64	128			64	128			>128	>128				>128	
Ae1		32–64	128	4 (8–16)	32 (1–2)	32–64	32–64	16 (2–4)	64 (1)	>128	>128	>128 (1)		>128 (1)	>128	>128
		MIC_50_ = 32, MBC_50_ = 64		MIC_50_ = 2, MBC_50_ = 32		MIC_50_ = 32, MBC_50_ = 64		MIC_50_ = 16, MBC_50_ = 64		MIC_5 0_> 128, MBC_50_ > 128		MIC_50_ > 128, MBC_50_ > 128				
		MIC_90_ = 64, MBC_90_ = 128		MBC_50_/MIC_50_ = 16		MIC_90_ = 64, MBC_90_ = 128		MBC_50_/MIC_50_ = 4		MIC_90_ > 128, MBC_90_ > 128						
		MBC_50_/MIC_50_ = 8				MBC_50_/MIC_50_ = 2				>128						
***P. multocida***																
CVCC1729	A:1,3	2	32	2 (1)	16 (2)	0.25	0.5	0.03 (8)	0.5 (1)	64	>128	32 (2)		32 (>4)	128	>128
CVCC2083	A:1,4	2	32	2 (1)	32 (1)	0.25	8	0.03 (8)	0.5 (16)	>128	>128	64 (>2)		128 (>1)	>128	>128
Ap1		4	16	1 (4)	16 (1)	0.13	1	0.03 (4)	0.5 (2)	128	>128	32 (4)		64 (>2)	128	>128
		MIC_50_ = 2, MBC_50_ = 32		MIC_50_ = 2, MBC_50_ = 16		MIC_50_ = 0.25, MBC_50_ = 1		MIC_50_ = 0.03, MBC_50_ = 0.5		MIC_50_ = 128, MBC_50_ > 128		MIC_50_ = 32, MBC_50_ = 64				
		MBC_50_/MIC_50_ = 16		MBC_50_/MIC_50_ = 8		MBC_50_ /MIC_50_ = 4		MBC_50_/MIC_50_ = 16				MBC_50_/MIC_50_ = 2				
***S. pullorum***																
Sa-s1		8	128	1 (8)	16 (8)	2	64	0.5 (4)	32 (2)	128	>128	128 (1)		>128 (1)	>128	>128
Sa-s2		8	128	1 (8)	16 (8)	2	64	0.5 (4)	32 (2)	64	>128	64 (1)		>128 (1)	>128	>128
Sa-h		8	128	1 (8)	16 (8)	128	>128	32 (4)	128 (2)	>128	>128	>128 (1)		>128 (1)	>128	>128
Sa-h2		8	128	1 (8)	16 (8)	2	64	0.5 (4)	64 (2)	128	>128	128 (1)		>128 (1)	>128	>128
Sa-x		16	128	2 (8)	32 (4)	2	64	0.5 (4)	32 (2)	128	>128	128 (1)		>128 (1)	>128	>128
Sa-p1		16	128	2 (8)	32 (4)	2	64	0.5 (4)	32 (2)	128	>128	64 (2)		>128 (1)	>128	>128
Sa-p2		32	128	2 (16)	32 (4)	2	32	0.5 (4)	32 (1)	128	>128	128 (1)		>128 (1)	>128	>128
X1		4	64			8	32			128	>128				>128	
		MIC_50_ = 8, MBC_50_ = 128		MIC_50_ = 1, MBC_50_ = 16		MIC_50_ = 2, MBC_50_ = 64		MIC_50_ = 0.5, MBC_50_ = 32		MIC_50_ = 128, MBC_50_ > 128		MIC_50_ = 128, MBC_50_ > 128				
		MIC_90_ = 16, MBC_90_ = 128		MIC_90_ = 2, MBC_90_ = 32		MIC_90_ = 8, MBC_90_ = 64		MIC_90_ = 0.5, MBC_90_ = 64		MIC_90_ = 128, MBC_90_ > 128		MIC_90_ = 128, MBC_90_ > 128				
		MBC_50_/MIC_50_ = 16		MBC_50_/MIC_50_ = 16		MBC_50_/MIC_50_ = 32		MBC_50_/MIC_50_ = 64								
***C. jejuni***																
ATCC BAA-1062™		0.25	1			0.13	0.5			128	128				>128	
		MBC/MIC = 4				MBC/MIC = 4				MBC/MIC = 1						
G ^+^																
***S. aureus***																
As1		16–32	64			0.13	4			32	32				>128	
As2		32	64			16	32			16	32				>128	
Z1		64	>128			32	>128			16	>128				>128	
		MIC_50_ = 32, MBC_50_ = 64				MIC_50_ = 16, MBC_50_ = 32				MIC_50_ = 16, MBC_50_ = 32						
		MBC_50_/MIC_50_ = 2				MBC_50_/MIC_50_ = 2				MBC_50_/MIC_50_ = 2						
***Enterococcus* spp.**																
CVCC1297	Gram-D group	64	>128	8 (8)	64 (>2)	16	128	8 (2)	32 (4)	32	128	32 (1)		64 (2)	>128	>128
CVCC1298	Gram-D group	64	>128	16 (4)	64 (>2)	0.5	4	0.5 (1)	4 (1)	64	>128	32 (2)		64 (>2)	>128	>128
		MBC/MIC > 2		MBC/MIC = 4–8		MBC/MIC = 8		MBC/MIC = 4–8		MBC/MIC = 4		MBC/MIC = 2				
***C. perfringens***																
CVCC2030	A	1	1			8	8			4	4				>128	
		MBC/MIC = 1				MBC/MIC = 1				MBC/MIC = 1						

Note: (1) The lower symbol “_S_” denotes the result was under CLSI condition, and “_N_” denotes the result was under anaerobic condition. (2) Values in brackets are the multiple drop of MIC under anaerobic conditions from aerobic conditions. (3) Includes *E. faecalis* (CVCC1297) and *E. faecium* (CVCC1298). “+” denotes gram-positive and “−” denotes gram-negative.

**Table 3:** Antimicrobial susceptibility of cyadox and controls against common pathogens isolated from fishes (unit: μg/mL).

Strains	Number	Cyadox				Chlortetracycline				Sulfadimidine				Dimethyl Sulfoxide	
		MIC_S_	MBC_S_	MIC_N_	MBC_N_	MIC_S_	MBC_S_	MIC_N_	MBC_N_	MIC_S_	MBC_S_	MIC_N_	MBC_N_	MIC_S_	MIC_N_
**G^-^**															
***E. coil***	Se1	1	16			2	4			1	2			128	128
***Y. ruckeri***	SC90-2-4	32	128	2 (16)	8 (16)	2	16	0.25 (8)	1 (16)	128	>128	>128 (1)	>128 (1)	128	128
***A. hydrophila***	XS91-4-1	64	128	1 (64)	2 (64)	0.5	0.5	0.13 (4)	0.25 (2)	>128	>128	64 (1)	>128 (1)	128	128
	Ah78	64	128	0.5 (128)	1 (128)	2	16	0.13 (16)	2 (32)	>128	>128	>128 (1)	>128 (1)	>128	128
	Ah561	128	128	0.5 (256)	1 (128)	0.5	8	0.13 (4)	2 (4)	>128	>128	>128 (1)	>128 (1)	>128	64
	Ah563	128	128	1 (128)	2 (64)	0.5	2	0.13 (4)	0.5 (4)	64	128	64 (1)	128 (1)	>128	128
	A1	64	128	0.5 (128)	1 (128)	0.25	2	0.13 (2)	0.5 (4)	128	128	128 (1)	>128 (1)	128	128
		MIC_50_ = 64, MBC_50_ = 128		MIC_50_ = 0.5, MBC_50_ = 1		MIC_50_ = 0.5, MBC_50_ = 2		MIC_50_ = 0.13, MBC_50_ = 0.5		MIC_50_ > 128, MBC_50_ > 128		MIC_50_ = 128, MBC_50_ > 128			
		MBC_50_/MIC_50_ = 2		MBC_50_/MIC_50_ = 2		MBC_50_/MIC_50_ = 4		MBC_50_/MIC_50_ = 4							
***A. veronii***	ATCC9071	64	128	0.5 (128)	4 (32)	≤0.25	1	0.13 (≤2)	0.13 (8)	>128	>128	>128 (1)	>128 (1)	128	128
***A. jandaei***	F30-3	64	128	2 (32)	4 (32)	≤0.25	1	0.06 (≤4)	0.13 (8)	>128	>128	64 (>2)	>128 (1)	128	128
***A. caviae***	DMA1-A	64	128	1 (64)	4 (32)	≤0.25	1	0.25 (1)	0.5 (2)	>128	>128	>128 (1)	>128 (1)	128	128
***A. punctata***	58-20-9	128	128	128 (1)	128 (1)	128	128	64 (2)	128 (1)	>128	>128	>128 (1)	>128 (1)	128	128
***A. sobria***	CR79-1-1	128	128	0.5 (256)	1 (128)	1	2	0.06 (16)	0.13 (16)	>128	>128	>128 (1)	>128 (1)	128	128
	3-6	128	128			0.5	2			>128	>128			>128	
	3-7	64	128			4	8			>128	>128			>128	
	3-8	128	128			4	8			>128	>128			>128	
		MIC_50_ = 128, MBC_50_ = 128				MIC_50_ = 1, MBC_50_ = 2				MIC_50_ > 128, MBC_50_ > 128					
		MBC_50_/MIC_50_ = 1				MBC_50_/MIC_50_ = 2									
***E. ictaluri***	HSN-1	64	64	64 (1)	64 (1)	4	4	≤0.03 (≥128)	1 (4)	>128	>128	>128 (1)	>128 (1)	128	128
***V. fluvialis***	WY91-24-3	64	128	0.5 (128)	2 (32)	0.5	8	0.13 (4)	0.25 (32)	>128	>128	>128 (1)	>128 (1)	128	128
***F. columnare***	G4	2	>128			0.5	16			8	128			64	
***A. baumannii***	Ab1	64	128	8 (8)	16 (8)	0.125	0.5	≤0.03(≥4)	0.13 (4)	16	32	16 (1)	32 (1)	128	128
***P. fluorescent***	W81-11	128	>128	128 (1)	>128 (1)	4	8	2 (2)	4 (2)	>128	>128	>128 (1)	>128 (1)	>128	128
	56-12-10	128	>128	128 (1)	128 (>1)	16	128	4 (4)	64 (2)	>128	>128	>128 (1)	>128 (1)	128	128
	1-1	128	>128			16	128			>128	>128			128	
	1-2	128	>128			16	128			>128	>128			128	
	1-3	128	>128			32	128			>128	>128			128	
	1-4	128	>128			16	128			>128	>128			128	
	1-5	128	>128			16	128			>128	>128			128	
	1-6	128	>128			16	128			>128	>128			128	
	1-7	64	>128			16	128			>128	>128			128	
	1-8	64	>128			32	128			>128	>128			128	
		MIC_50_ = 128, MBC_50_>128				MIC_50_ = 16, MBC_50_ = 128				MIC_50_ > 128, MBC_50_ > 128					
		MIC_90_ = 128, MBC_90_ > 128				MIC_90_ = 32, MBC_90_ = 128				MIC_90_ > 128, MBC_90_ > 128					
						MBC_50_/MIC_50_ = 8									
**G^+^**															
***S. aureus***	Fs1	64	128			0.125	0.5			16	128			128	
	Fs2	16	64			0.125	1			>128	>128			>128	
***S. agalactiae***	XQ-1	16	32	8 (2)	16 (2)	0.125	2	≤0.03 (2)	0.13 (16)	>128	>128	>128 (1)	>128 (1)	>128	128
***M. tuberculosis***	Asc-1.2Ⅱ	16	32			8	32			>128	>128			>128	
	Asc-1.3Ⅱ	32	64			32	64			>128	>128			>128	
	Asc-1.3Ⅴ	32	64			32	128			>128	>128			>128	
	Cst-t-10	32	64			>128	>128			>128	>128			>128	
		MIC_50_ = 32, MBC_50_ = 64				MIC_50_ = 32, MBC_50_ = 64				MIC_50_>128, MBC_50_>128					
		MBC_50_/MIC_50_ = 2				MBC_50_/MIC_50_ = 2									

Note: (1) The lower symbol “_S_” denotes the result was under CLSI condition, and “_N_” denotes the result was under anaerobic condition. (2) Values in brackets are the multiple drop of MIC under anaerobic conditions from aerobic conditions. “+” denotes gram-positive and “−” denotes gram-negative.

**Table 4:** Antimicrobial susceptibility of cyadox and controls against pathogens isolated from others (unit: μg/mL).

Numbers of Strains	Serotype	Cyadox				Chlortetracycline				Olaquindox					Bacitracin zinc				Dimethyl Sulfoxide		
		MIC_S_	MBC_S_	MIC_N_	MBC_N_	MIC_S_	MBC_S_	MIC_N_		MBC_N_	MIC_S_	MBC_S_	MIC_N_	MBC_N_		MIC_S_	MIC_N_	MIC_S_	MIC_N_	MIC_S_	MIC_N_
**G^-^**																					
***S. typhimurium***																					
CVCC542	1,4,12:i:1,2	8	>128	8 (1)	64 (2)	8	128	0.5(16)	≥64 (1)	32	64	2 (16)	16 (4)		>128	>128	>128 (1)		>128 (1)	>128	>128
***Y. enterocolitica***																					
ATCC 9610 ^TM^	Group, O:8	32	128	16 (2)	64 (2)	1	4	0.5 (2)	8 (1)	8	16	4 (2)	16 (1)		-		>128 (1)		>128 (1)	>128	128
***P. mirabilis***																					
ATCC 29245^TM^		32	128	4 (8)	32 (4)	>128	>128	64 (>2)	128 (>1)	16	32	2 (8)	4 (8)		-		>128 (1)		128	>128	128
***P. pyocyanea***																					
CVCC2087		128	>128	128 (1)	>128 (1)	32	128	8 (4)	32 (4)	>128	>128	64 (>2)	128 (>1)		>128	>128	128 (1)		>128 (1)	>128	>128

Note: (1) The lower symbol “_S_” denotes the result was under CLSI condition, and “_N_” denotes the result was under anaerobic condition. (2) Values in brackets are the multiple drop of MIC under anaerobic conditions from aerobic conditions. “+” denotes gram-positive and “−” denotes gram-negative.
